# Electroencephalography and Functional Magnetic Resonance Imaging-Guided Simultaneous Transcranial Direct Current Stimulation and Repetitive Transcranial Magnetic Stimulation in a Patient With Minimally Conscious State

**DOI:** 10.3389/fnins.2019.00746

**Published:** 2019-07-31

**Authors:** Yicong Lin, Tiaotiao Liu, Qian Huang, Yingying Su, Weibi Chen, Daiquan Gao, Xin Tian, Taicheng Huang, Zonglei Zhen, Tao Han, Hong Ye, Yuping Wang

**Affiliations:** ^1^Department of Neurology, Xuanwu Hospital, Capital Medical University, Beijing, China; ^2^School of Biomedical Engineering, Tianjin Medical University, Tianjin, China; ^3^State Key Laboratory of Cognitive Neuroscience and Learning and IDG/McGovern Institute for Brain Research, Beijing Normal University, Beijing, China; ^4^The Beijing Key Laboratory of Neuromodulation, Beijing, China; ^5^Center of Epilepsy, Beijing Institute for Brain Disorders, Capital Medical University, Beijing, China

**Keywords:** minimally conscious state, simultaneous stimulation, repetitive transcranial magnetic stimulation, transcranial direct current stimulation, functional magnetic resonance imaging

## Abstract

**Objective:**

A minimally conscious state (MCS) is characterized by discernible behavioral evidence of consciousness that cannot be reproduced consistently. This condition is highly challenging to treat. Recent studies have demonstrated the potential therapeutic effect of non-invasive brain stimulation in patients with MCS. In one patient in an MCS, we delivered simultaneous transcranial direct current stimulation (tDCS) and repetitive transcranial magnetic stimulation (rTMS) based on an individual brain network analysis and evaluated the therapeutic effect.

**Methods:**

The directional transfer function (DTF) was calculated based on electroencephalograph (EEG) analysis. Global brain connectivity was calculated based on functional magnetic resonance imaging (fMRI) analysis. By referring to the EEG and fMRI results, we identified inferior parietal lobes (IPLs) as targets. In the 2-week treatment period, 14 sessions were applied to the identified bilateral parietal regions. Simultaneous 1.5-mA anodal tDCS and 5-Hz rTMS were delivered for 20 min per hemisphere in each session. Clinical evaluation scores were recorded weekly throughout the treatment. A second patient given the routine treatment was evaluated as a control.

**Results:**

The clinical scores of patient 1 with MCS improved after 2 weeks of stimulation treatment, and the effect lasted for up to 1 month. EEG analysis showed a significant increase (*p* < 0.001) in the DTF value in the gamma band in a bilateral set of posterior regions, and fMRI showed a trend toward normalized activity in the IPLs. The clinical scores of patient 2 with coma did not improve much after 2 weeks of routine treatment. The EEG analysis showed a significant increase (*p* = 0.021) in the DTF value in the gamma band in a bilateral set of posterior regions.

**Conclusion:**

The application of EEG and fMRI to characterize the functional connectivity features of the network in an MCS patient provided a reasonable and accurate stimulation target and verified the changes in functional connectivity resulting from stimulation.

## Introduction

A minimally conscious state (MCS) is characterized by discernible behavioral evidence of consciousness that cannot be reproduced consistently (Giacino et al., 2002). An MCS may result from degenerative nervous system disorders or evolve from a coma or vegetative state (VS). In MCS patients, integrated cortical functions are retained but undersustained. MCS is a highly challenging clinical condition to treat. Notably, recent studies have demonstrated the potential therapeutic effect of non-invasive brain stimulation (NIBS) in patients with MCS.

Repetitive transcranial magnetic stimulation (rTMS) and transcranial direct current stimulation (tDCS) are two NIBS techniques that have been developed in the past decades. High-frequency rTMS can decrease GABAergic activity and decrease synaptic transmission through a long-term potentiation-like mechanism (Lefaucheur et al., 2014). Anodal tDCS can change the resting membrane potential by influencing ion channels and gradients, thus increasing cortical excitability (Lefaucheur et al., 2017). These techniques have recently been employed independently in the treatment of MCS and have shown some inspiring beneficial results.

Several studies have shown that priming stimulation can alter the effect of a test stimulation on cortical excitability. Nitsche et al. (2007) found that cortical excitability was higher with simultaneous anodal tDCS and single-pulse TMS than with anodal tDCS alone. We hypothesized that a protocol of simultaneous anodal tDCS and high-frequency rTMS would produce an enhanced excitatory effect. The actual background network activity was predicted to boost the expected effect of rTMS (Muller-Dahlhaus and Ziemann, 2015).

To maximize the benefit of NIBS treatment in a patient with MCS, we designed a simultaneous stimulation protocol based on an individual brain network analysis. We identified stimulation targets and evaluated therapeutic effects by analyzing functional connections based on scalp electroencephalography (EEG) and functional magnetic resonance imaging (fMRI). As a control, we also evaluated another patient with similar structural damage who did not receive stimulation.

## Materials and Methods

### Patients

Patient 1 was diagnosed with MCS according to the Coma Recovery Scale-Revised (CRS-R) (Giacino et al., 2004) due to brain stem hemorrhage 1 month prior. The patient could blink his eyes and move after stimulus, but could not consistently move to auditory command. His eyes could move from the initial target to a new target for more than 2 s. There was not any vocalization or oromotor movement. There was some discernable non-verbal communication response. On neurological examination, the pupils were equal in size and reactive to light. Corneal reflexes and gag reflexes were bilaterally present. The Babinski sign was positive bilaterally. Brain magnetic resonance imaging (MRI) showed subacute hemorrhage in the pons, left cerebral peduncle, and brachium pontis. Before stimulation, the patient’s CRS-R score was 10 points based on auditory function (3), visual function (2), motor function (3), verbal function (0), communication (1), and arousal (1) criteria. The Glasgow Coma Scale (GCS) score was 10 based on eye (3), verbal (1), and motor (6) criteria. The Full Outline of UnResponsiveness (FOUR) score was 15 points. The Coma/Near Coma Scale (CNC) score was 18 points. The patient received gangliosides, ambroxol, imipenem, valsartan, captopril, and enteral nutritional suspension. The demographics, clinical data, and EEG analysis results of patient 1 are shown in detail in Table 1.

**Table 1:** Detailed demographics, clinical data, and EEG analysis results of the two patients studied.

No.	Age	Time to injury	Imaging characteristics	Current clinical state based on physical and standardized evaluations	Medication	Clinical EEG findings
1	31	1 month	MRI showed subacute hemorrhage in the pons, left cerebral peduncle and brachium pontis.	The pupils were equal in size and reactive to light. Corneal reflexes and gag reflexes were bilaterally present. The Babinski sign was positive bilaterally. The patient spontaneously blinked his eyes infrequently. The patient could blink his eyes after pain stimulus, but could not consistently move to acoustic command. His eyes could move from the initial target to a new target for more than 2 s. There was not any vocalization or oromotor movement.	Gangliosides, ambroxol, imipenem, valsartan, captopril, and enteral nutritional suspension	Continuous diffused slow (5–7 Hz), infrequently intermittent slow (2–4 Hz), infrequent spindle, non-reactive to stimulus.
2	83	1 week	CT showed hemorrhage in the mid brain and pons.	The pupils were equal in size and slowly reactive to light. The corneal reflex was present on the left but diminished on the right. The Doll’s head eye phenomenon was not present. The Babinski sign was positive on the right side. The patient did not respond well to stimulus except for some withdraw movement to pain stimulus and avoidance to light stimulus.	Edaravone, mannitol, amlodipine, ampenem, vancomycin, reduced glutathione, ambroxol, famotidine, and enteral nutritional suspension.	Continuous diffused slow (5–7 Hz), frequently intermittent slow (2–4 Hz), non-reactive to stimulus.


*MRI, magnetic resonance imaging; EEG, electroencephalogram; CT, computed tomography.*

Patient 2 was diagnosed with coma due to brain stem hemorrhage 1 week prior. At baseline, the patient did not respond well to stimulus except for some withdraw movement to pain stimulus and avoidance to light stimulus. On neurological examination, the pupils were equal in size and slowly reactive to light. The corneal reflex was present on the left but diminished on the right. The Doll’s head eye phenomenon was not present. The Babinski sign was positive on the right side. Brain computed tomography (CT) showed hemorrhage in the mid brain and pons. At the baseline, the patient’s CRS-R score was 3 points based on auditory function (0), visual function (1), motor function (2), verbal function (0), communication (0), and arousal (0) criteria. The GCS score was 6 based on eye (1), verbal (1), and motor (4) criteria. The FOUR score was 5 points. The CNC score was 22 points. The patient received edaravone, mannitol, amlodipine, ampenem, vancomycin, reduced glutathione, ambroxol, famotidine, and enteral nutritional suspension. The demographics, clinical data, and EEG analysis results of patient 2 are shown in detail in Table 1.

### EEG Analysis

EEG analysis was performed based on 1-h 32-channel scalp EEG data. The EEG signals were offline low-pass-filtered (100 Hz) and notch-filtered (49–51 Hz) and baseline correction was performed though polynomial fitting. Eye movements and significant muscle artifacts were also excluded with Automatic Artifact Removal toolbox (Gómez-Herrero, 2007). The signal-to-noise ratio (SNR) of the signal in channel i is defined as: $SNR_{i}=\frac{\sigma_{signal(i)}^{2}}{\sigma_{noise}^{2}}$, where σ_*signal*(*i*)_ denotes the standard deviation of the EEG signal in channel i and σ_*noise*_ denotes the standard deviation of the noise signal. The noise signal is estimated using the standard deviation of the pre interval (Zhang et al., 2016). The SNRs of the signals among channels after preprocessing ranged from 7 to 10, therefore ensuring the robustness of the following causality analysis (Fasoula et al., 2013). In the framework of the multivariate autoregressive (MVAR) model, multichannel EEGs can be described as a data vector X of N source signals: *X*(*t*) = {*x*_1_(*t*), *x*_2_(*t*),..., *x*_*N*_(*t*)}.

The MVAR model can then be constructed as follows:

(1) $$X(t)=\sum_{n=1}^{p}A_{n}X(t-n)+E(t)$$

where *E*(*t*) is a vector of multivariate zero-mean uncorrelated white noise at time *t, A_n_* is an N × N matrix of the model coefficients, and *p* is the model order. In the present study, the model order was calculated though the ARFIT package in eConnectome toolbox (Supplementary Table 1). As order selection criteria, ARFIT computes approximations to Schwarz’s Bayesian Criterion (SBC) and to the logarithm of Akaike’s Final Prediction Error (He et al., 2011). The MVAR model was then transformed into the frequency domain:

(2) $$X(f)=A^{-1}(f)E(f)=H(f)E(f)$$

where *f* denotes a specific frequency and the *H*(*f*) matrix is the transfer matrix defined as follows:

(3) $$H(f)=A^{-1}(f)={\left(\sum_{i=0}^{p}A(i)e^{-j2\pi fi\Delta t}\right)}^{-1},\;A_{0}=-I$$

where *I* is an identity matrix.

The directional transfer function (DTF) is defined by the elements of the transfer matrix *H_ij_* as follows:

(4) $$\gamma_{ij}{\left(f\right)}^{2}=|H_{ij}{|}^{2}/\sum_{m=1}^{N}|H_{im}(f){|}^{2}$$

where γ_*ij*_(*f*) expresses the ratio between inflow from node *j* to node *i* and all inflows to node *i*, and *N* is the number of nodes. Once the causal interactions from the DTF calculation for the analyzed epoch were obtained, statistical significance testing was performed to remove the links that formed spurious interactions between EEG channels. A surrogate data method was applied to each analyzed epoch in which the temporal correlation between the EEG channels was destroyed. The shuffling and connectivity estimation procedures were repeated a certain number of times (e.g., 1,000), yielding a distribution of the DTF values under the null hypothesis that no connectivity exists. Based on this empirical distribution, the critical value of significance was set at *p* < 0.05. The statistical assessment procedure was implemented for connectivity estimation to obtain real causal interactions. The DTF values among EEGs were calculated and converted into a DTF matrix (Wilke et al., 2011; Zhang et al., 2016). The DTF value is a function of frequency, which covers the major MCS rhythms. Therefore, the mean value of all the elements in the DTF matrix (DTF_mean_) is a direct measurement of functional connectivity strength among EEGs. The results of the DTF values across different ranges have been added in the Supplementary Materials (Supplementary Figure 1).

### fMRI Analysis

For fMRI analysis, we used a global brain connectivity (GBC) method (Cole et al., 2012; Wang et al., 2016) to characterize the averaged connectivity of each voxel to the rest of the voxels in the default mode network (DMN) or executive control network (ECN), which were defined from Yeo et al. (2011). The GBC method was performed by calculating the functional connectivity (i.e., correlation) of a voxel in the DMN or ECN to the rest of the voxels, one by one, and the functional connectivity was then averaged as the connectivity of each voxel to produce a GBC value. This method takes advantage of characterizing overall functional connectivity with voxelwise resolution, enabling us to examine the impairment of each network’s functional connectivity in one patient.

### Target Identification

**FIGURE 1:**
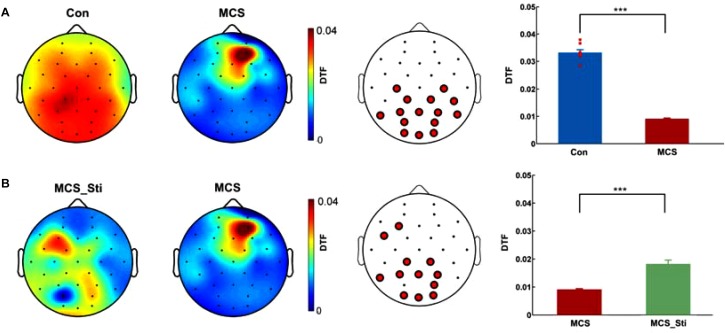
Comparison of DTF values in the gamma frequency of EEGs between controls and patient 1 (MCS) before and after stimulation treatment. **(A)** DTF spatial distribution in healthy controls (Con) and patient 1 (MCS). The color bar denotes the DTF values. First panel: eight healthy controls. Second panel: compared to the eight healthy controls, the MCS patient showed a significantly decreased DTF value in the gamma frequency in bilateral posterior regions. Third panel: 15 electrodes with significantly reduced DTF values in patient 1 compared to those in healthy controls were identified. Fourth panel: the average DTF value of the 15 electrodes was significantly lower in the MCS patient than in Con (^∗∗∗^*p* < 0.001, *t*-test/Wilcoxon’s rank sum test). The blue bar denotes the DTF value of the 15 electrodes in eight controls. The red bar denotes the pretreatment DTF value of the 15 electrodes in the patient. The DTF value (averaged across channels) of each single healthy control is shown as a dot. **(B)** Spatial distribution of the DTF value in patient 1 before and after treatment. First panel: the MCS patient after stimulation treatment showed a significant increase in the DTF value in the gamma frequency in bilateral posterior regions. Second panel: the MCS patient before treatment. Third panel: 12 electrodes with significantly higher DTF values after treatment compared to those before treatment were identified (^∗∗∗^*p* < 0.001). The red bar denotes the pretreatment DTF value of the 12 electrodes in the patient. The green bar denotes the post-treatment DTF value of the 12 electrodes in the patient. DTF, directional transfer function; EEG, electroencephalography; Con, controls; MCS, minimally conscious state; MCS_Sti, minimally conscious state after treatment.

**FIGURE 2:**
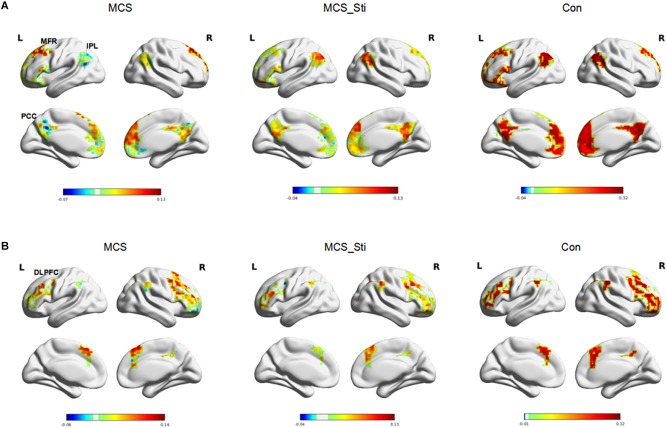
GBC analysis of fMRI in controls (Con) and patient 1 (MCS) before and after stimulation treatment. **(A)** DMN network. First panel: before treatment, patient showed decreased activity in regions, including the IPL, MFR, and PCC. Second panel: after treatment, patient 1 showed an increase in IPL and PCC activity. Third panel: healthy controls. **(B)** ECN network. First panel: before treatment, patient 1 showed decreased activity in the DLPFC. Second panel: after treatment, patient 1 showed a decrease in DLPFC activity. Third panel: healthy controls. GBC, global brain connectivity; fMRI, functional magnetic resonance imaging; DMN, default mode network; ECN, extrinsic control network; Con, controls; MCS, minimally conscious state; MCS_Sti, minimally conscious state after treatment; MFR, mesiofrontal region; IPL, inferior parietal lobe; PCC, posterior cingulate cortex; DLPFC, dorsolateral prefrontal cortex.

**FIGURE 3:**
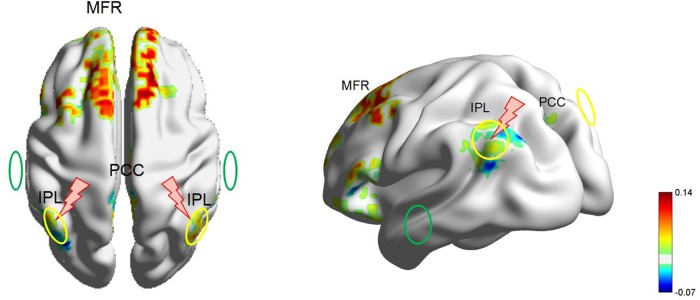
The locations of the rTMS and tDCS targets in a head model of patient 1. The yellow circle denotes the anode electrode over P3 or P4. The green circle denotes the cathode electrode over T3 or T4. The red lightning mark denotes the rTMS site over P3 or P4. For the 20-min treatment on the right hemisphere, the anodal tDCS was delivered over P4, and the cathodal tDCS was delivered over T3. Meanwhile, 5-Hz rTMS was delivered precisely over the anodal electrode at P4. Then, for the next 20-min treatment on the left hemisphere, the anodal tDCS was delivered over P3, and the cathodal tDCS was delivered over T4. Meanwhile, 5-Hz rTMS was delivered precisely over the anodal electrode at P3. rTMS, repetitive transcranial magnetic stimulation; tDCS, transcranial direct current stimulation; fMRI, functional magnetic resonance imaging; IPL, inferior parietal lobe; PCC, posterior cingulate cortex; MFR, mesiofrontal region.

Compared to eight healthy controls, patient 1 presented a significant decrease in the DTF value in gamma frequency in the bilateral posterior regions, as shown in Figure 1. These 15 decreased electrodes included bilateral occipital (O1, O2, Oz in 10–20 International Electrode System), parieto-occipital (PO3, PO4), parietal (P3, P4, Pz), centro-parietal (CP1, CP2), central (C3, C4), posterior temporal (T5, T6), and right centro-temporal (CP6) regions. In resting fMRI analysis, we found a profound visual decrease in brain functional connectivity in regions of the DMN, including the inferior parietal lobe (IPL), posterior cingulate cortex (PCC), and mesiofrontal region (MFR), as well as regions of the ECN, including the dorsolateral prefrontal cortex (DLPFC), as shown in Figure 2. Herwig et al. (2003) studied the corresponding cortical sites of the 10–20 International Electrode System using neuronavigation and found that targeting P3 mainly reached BA 40 and to a lesser extent BA 7 in the inferior part of the parietal lobe. Therefore, to better cover common regions identified by EEG and fMRI, we identified bilateral parietal regions (P3 and P4) as anodal tDCS targets and contralateral temporal regions (T4 and T3) as cathodal tDCS targets, and P3 and P4 as high-frequency TMS targets. Figure 3 shows these targets in the individual head model.

The electric field distribution in the brain was simulated using SimNIBS 2.1.1 software (Thielscher et al., 2015). The simulation was generated based on the template head model included in the software package. We simulated the electric field distribution of the anode at P4 with the cathode at Fp1 and the anode at P4 with T3. The simulated electric field was more restricted when the anode is P4 and the cathode is T3. We also simulated the magnetic field when the target was set at P4. The simulated magnetic field and electric field were consistent.

### Stimulation Protocol

For patient 1, the 14 sessions of simultaneous anodal tDCS and high-frequency rTMS were delivered over the course of 2 weeks, and a clinical evaluation was performed weekly throughout the course of treatment. Both electrical and magnetic stimulation were delivered using an Electromagnetic Stimulator (Yunshen Technology Limited Company, Beijing, China). Direct current was delivered by a pair of saline-soaked silver cloth-wrapped sponge electrodes (thickness, 0.4 cm; area of electrode, 7 cm^2^, Greentek, Pty Ltd., China). rTMS was delivered through a circular coil (diameter, 74 mm; peak magnetic field, 2.0 Tesla). Forty minutes of stimulation (20 min for each hemisphere) were given per day. For the 20-min treatment on the right hemisphere, anodal tDCS was delivered over P4, and cathodal tDCS was delivered over T3. Meanwhile, 5 Hz rTMS was delivered precisely over the anodal electrode at P4. The current was ramped up to 1.5 mA (for 10 s) from the onset of stimulation, applied for 20 min, and ramped down to 0 mA (for 10 s). One rTMS train consisted of 25 pulses delivered at 5 Hz, with an intertrain interval of 55 s. In a single session, 500 pulses (20 rTMS trains) were delivered for 20 min. The strength of the stimulation was 70% of the resting motor threshold (RMT). Then, for the next 20-min treatment on the left hemisphere, anodal tDCS was delivered over P3, and cathodal tDCS was delivered over T4. Meanwhile, 5 Hz rTMS was delivered precisely over the anodal electrode at P3.

## Results

### Clinical Assessments

**FIGURE 4:**
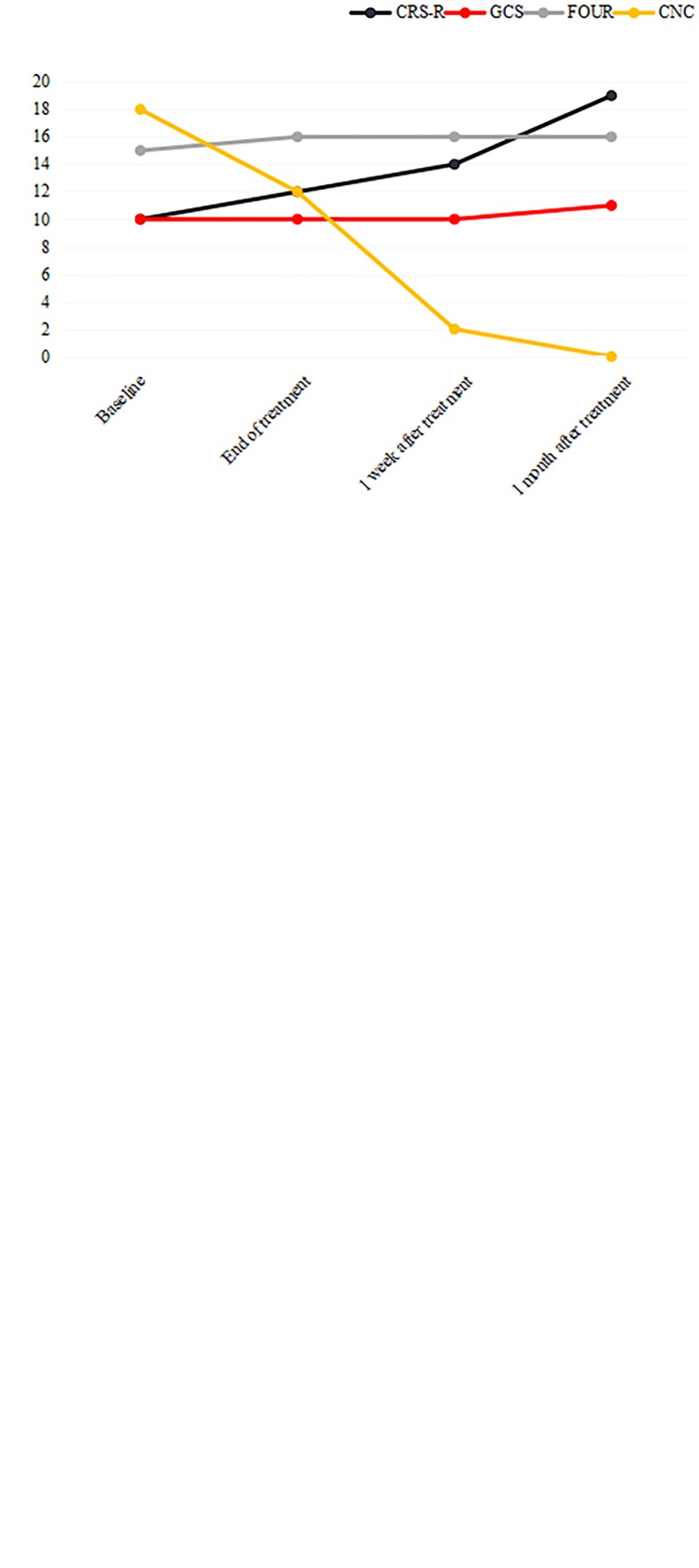
Clinical scores before, during, and after treatment in patient 1 and patient 2. **(A)** The CRS-R, GCS, FOUR, and CNC scores in patient 1. Compared to baseline, the CRS-R and CNC scores improved considerably, and the effect lasted for up to 1 month. **(B)** The subscales of the CRS-R score in patient 1. The arousal and auditory functions were the first to show improvement during stimulation. Then, communication improved. Finally, visual function and motor function improved after stimulation treatment was completed. **(C)** The CRS-R, GCS, FOUR, and CNC scores in patient 2. Compared to baseline, the scores did not show much improvement after 2 weeks of treatment. CRS-R, Coma Recovery Scale-Revised; GCS, Glasgow Coma Scale; FOUR, Full Outline of UnResponsiveness; CNC, Coma/Near Coma Scale.

During stimulation treatment, patient 1 could move to acoustic command consistently, and his eyes could open and track after verbal prompt. After stimulation, the patient improved gradually. He could recognize objects and could give consistent behavioral response to verbal prompt. The patient could spontaneously open eyes, track and fix, and could open mouth when a spoon is near. The patient had discernable non-verbal communication response. The physical examinations did not change throughout the study. The patient was evaluated on a weekly basis through these four scales. At the end of the stimulation treatment period, the CRS-R, GCS, FOUR, and CNC were 12, 10, 16, and 12 points, respectively. At 1 week after treatment, the CRS-R, GCS, FOUR, and CNC were 14, 10, 16, and 2, respectively. At 1 month after treatment, the CRS-R, GCS, FOUR, and CNC were 19, 11, 16, and 0, respectively, as shown in Figure 4A and Table 2. Further analysis of subscales of the CRS-R score in patient 1 showed that the arousal and auditory functions were the first to show improvement during stimulation (Figure 4B). Then, communication improved. Finally, visual function and motor function improved after stimulation treatment was completed.

**Table 2:** Clinical scores of the two patients throughout the study.

No.	Scale type	Baseline	1 week of treatment	2 weeks of treatment	1 week after treatment	2 weeks after treatment	3 weeks after treatment	4 weeks after treatment
1	CRS-R	10	11	12	14	17	19	19
	GCS	10	10	10	10	11	11	11
	FOUR	15	15	16	16	16	16	16
	CNC	18	14	12	2	0	0	0
2	CRS-R	3	3	4	–	–	–	–
	GCS	6	5	6	–	–	–	–
	FOUR	5	5	5	–	–	–	–
	CNC	22	22	26	–	–	–	–


*CRS-R, Coma Recovery Scale-Revised; GCS, Glasgow Coma Scale; FOUR, Full Outline of UnResponsiveness; CNC, Coma/Near Coma Scale.*

Patient 2 was provided with routine treatment (without stimulation) and did not show much clinical improvement. The patient could not avoid light stimuli like before. The physical examinations did not change throughout the study. The patient was also evaluated on a weekly basis through these four scales. At 1 week, the CRS-R, GCS, FOUR, and CNC were 3, 5, 5, and 22, respectively. At 2 weeks, the CRS-R, GCS, FOUR, and CNC were 4, 6, 5, and 26, respectively, as shown in Figure 4C and Table 2.

### Brain Network Analysis Based on EEG

Patient 1 exhibited a significantly lower DTF value in the gamma frequency in the bilateral posterior regions than healthy controls, as shown in Figure 1. Fifteen electrodes, which included O1, O2, Oz, PO3, PO4, P3, P4, Pz, CP1, CP2, C3, C4, T5, T6, and CP6, showed significantly reduced DTF values (^∗∗∗^*p* < 0.001, *t*-test/Wilcoxon’s rank sum test) compared to those regions in healthy controls. After 2 weeks of stimulation, EEG analysis showed a significant increase in DTF in the gamma frequency in 12 electrodes (^∗∗∗^*p* < 0.001) from before treatment. These 12 electrodes included F3, FC5, CP1, CP2, T5, P3, Pz, P4, PO4, O1, Oz, and O2.

**FIGURE 5:**
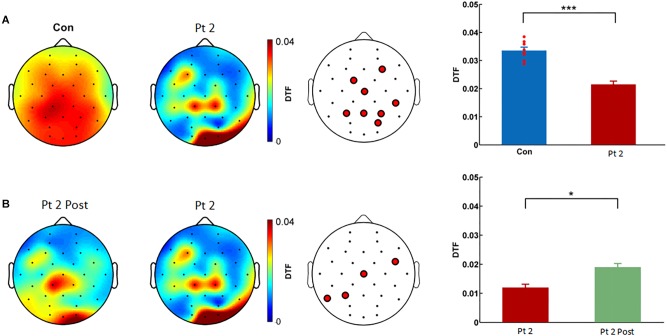
Comparison of DTF in the gamma frequency of EEG between controls and patient 2 before and after routine treatment. **(A)** DTF spatial distribution in healthy controls (Con) and patient 2. First panel: eight healthy controls. Second panel: compared to the eight healthy controls, patient 2 showed a significantly decreased DTF value in the gamma frequency in bilateral posterior regions. Third panel: eight electrodes (F4, FC1, Cz, P3, Pz, P4, PO4, and CP6) with significantly reduced DTF values in patient 2 compared to those in Con were identified. Fourth panel: the average DTF value of the eight electrodes was significantly lower in the patient 2 than in the Con (^∗∗∗^*p* < 0.001, *t*-test/Wilcoxon’s rank sum test). The blue bar denotes the DTF value of eight electrodes in eight controls. The red bar denotes the pretreatment DTF value of eight electrodes in the patient. The DTF value (averaged across channels) of each single healthy control is shown as a dot. **(B)** Spatial distribution of the DTF in patient 2 before and after routine treatment. First panel: after routine treatment, patient 2 showed a significant increase in the DTF value in the gamma frequency in bilateral posterior regions. Second panel: patient 2 before treatment. Third panel: four electrodes (FC6, Cz, P3, and T5) with significantly higher DTF values (*p* = 0.021) after treatment compared to those before treatment were identified (^∗^*p* < 0.1). The red bar denotes the pretreatment DTF value of the four electrodes in the patient. The green bar denotes the post-treatment DTF value of four electrodes in the patient. DTF, directional transfer function; EEG, electroencephalography; Con, controls; Pt 2 Post, patient 2 after treatment.

In patient 2, at baseline, EEG analysis showed a significantly lower DTF value in the gamma frequency in the bilateral posterior regions than in controls, as shown in Figure 5. Eight electrodes, including F4, FC1, Cz, P3, Pz, P4, PO4, and CP6, showed significantly reduced DTF values (^∗∗∗^*p* < 0.001). After 2 weeks of routine treatment, EEG analysis showed a significant increase in DTF in gamma frequency in four electrodes (*p* = 0.021) from baseline. These four electrodes included FC6, Cz, P3, and T5.

### Brain Network Analysis Based on fMRI

In patient 1, the DMN identified by baseline fMRI showed decreased activity in the IPL, PCC, and MFR (shown in Figure 2) compared to that in healthy controls. After stimulation treatment, there was a trend toward a normalization of IPL and PCC activity due to an increase in activity. The ECN identified by baseline fMRI showed decreased activity in the DLPFC (shown in Figure 2) compared to that in healthy controls. After stimulation treatment, there was no trend toward a normalization of DLPFC activity or increased activity.

## Discussion

This is an exploratory study in which an MCS patient received simultaneous tDCS and rTMS treatment based on brain network analysis of both EEG and fMRI. rTMS with 5–20 Hz and 90–100% of RMT and tDCS with 1–2 mA have been commonly utilized in previous studies (Lefaucheur et al., 2014, 2017). We utilized rTMS with 5 Hz and 70% of the RMT, and tDCS with 1.5 mA for safety and tolerability consideration. During stimulation, the MCS patient tolerated intervention well without displaying irritability and aggressiveness during head positioning or TMS/tDCS delivery. Clinical assessment showed improvement in the MCS patient. Another patient with similar structural damage was evaluated as a control and did not show much clinical improvement. Moreover, brain network analysis based on EEG and fMRI played important roles in this study. First, these tools were used to identify stimulation targets, potentially leading to more precise modulation. In addition, post-treatment analysis of the brain network was used to evaluate the treatment effect. We speculate that brain network-guided simultaneous tDCS and rTMS could be a promising treatment strategy for MCS.

### IPL Modulation Helps Treat MCS

The stimulation target chosen in this study was the bilateral IPL based on combined functional and electrophysiological datasets. In patient 1, there was a significant decrease in the DTF value in the bilateral centro-parieto-occipital regions identified by EEG analysis and decreased activity in the IPLs identified by fMRI analysis. After stimulation treatment, the patient achieved obvious improvement in clinical assessment and increased activity in bilateral IPLs. The IPL is involved in the DMN, which is related to self-awareness (Greicius et al., 2003; Tian et al., 2007) and shows decreased brain activity during loss of consciousness (Di Perri et al., 2016). Positron emission computed tomography studies have shown that neuronal activity in DMN regions increases upon recovery from VS (Laureys et al., 2006). Vanhaudenhuyse et al. (2010) observed a correlation between DMN integrity and the level of consciousness. Their group found that the DMN integrity decreased when descending from normal consciousness to MCS, VS, and coma, and the authors suggested that the connective strength of the PCC within the DMN can distinguish between VS and MCS patients. The functional connections within the DMN in MCS may reflect the chance of recovery. Different connectivity patterns could influence the efficacy of tDCS in MCS patients (Cavaliere et al., 2016). The re-establishment of functional connections within DMN regions may reflect the recovery of consciousness (Laureys et al., 2005).

However, in a literature review, previously reported targets are mostly in the DLPFC, motor cortex, orbitofrontal cortex, or a parieto-occipital region (Lefaucheur et al., 2014, 2017), rather than the IPL. The DLPFC is involved in the functional ECN, which is related to external awareness (D’Esposito et al., 1998; Lieberman, 2007). In addition to the DMN, the ECN has also been demonstrated to be altered in disorders of consciousness (Vanhaudenhuyse et al., 2011; Crone et al., 2014) and restored with the recovery of consciousness (Laureys and Schiff, 2012). In our study, we further analyzed the ECN in patient 1 and found decreased activity in the bilateral DLPFC when compared with that in healthy controls. Therefore, we suspected that the patient may also benefit from stimulation over the DLPFC.

The DMN and ECN are anticorrelated to each other under normal physiological conditions. However, these two networks are hypoactivated in patients with MCS in our study, potentially reflecting a reduction in the anticorrelation. The anticorrelated pattern has been shown to be of functional importance to the state of consciousness. The dynamics of the anticorrelation between the ECN and DMN during MCS have not been well-clarified. Heine et al. (2012) suspected that the anticorrelation generally diminishes or even disappears during conditions of altered consciousness. A stronger anticorrelation between the ECN and DMN has been shown to potentially reflect a better capacity to switch between internal and external modes of attention, which is necessary for maintaining conscious awareness (Leech et al., 2011; Di Perri et al., 2016). Anatomically, the superior longitudinal fascicle (SLF) connects the parietal cortex with the frontal cortex, and SLF II shows a strong connectivity to the DLPFC from the IPL (Parlatini et al., 2017). These results probably explain the accordant effect of excitatory stimulation of either the IPL or DLPFC, although further evidence is warranted.

### The Synergistic Effect of tDCS and rTMS

In previous studies, the efficacy of NIBS for MCS has been mild to moderate and variable in patients. A study explored the effect of high-frequency rTMS over the motor cortex in six patients with MCS or VS and found reappearance of fast activity and an increase in slow activity upon EEG analysis and behavioral changes in one patient with MCS (Manganotti et al., 2013). A case series trial found that patients with MCS can benefit from anodal tDCS over the left DLPFC (Angelakis et al., 2014). Three randomized controlled trials of tDCS in patients with MCS showed moderately improved recovery of signs of consciousness after anodal tDCS over the left DLPFC (Thibaut et al., 2014, 2017; Martens et al., 2018).

In this study, a simultaneous combination of tDCS and rTMS was explored. The patient showed a clinical improvement and a trend toward a normalization of functional connectivity. We speculate that the simultaneous tDCS and rTMS protocol produces beneficial synergistic effects, although we can only speculate on the underlying cellular and molecular mechanisms of these synergistic effects. A candidate mechanism might be the consolidation of long-term potentiation by protein synthesis and gene transcription (Muller-Dahlhaus and Ziemann, 2015). Muller-Dahlhaus and Ziemann (2015) found that either non-homeostatic metaplasticity or homeostatic metaplasticity occurred depending on successive NIBS protocols. Non-homeostatic metaplasticity can increase NIBS-induced aftereffects on cortical excitability. We speculate that non-homeostatic metaplasticity also prevailed when tDCS and rTMS were delivered simultaneously. The non-homeostatic metaplasticity may be explained by the assumption that tDCS and TMS activate neuronal circuits without a significant physiological interaction; therefore, the resulting effect reflects an arithmetic summation of the electrical and magnetic effects (Nitsche et al., 2007).

### Limitations

This is a preliminary two-case study. The patient with stimulation was in an MCS, and whether this treatment can be used in patients with other disorders of consciousness needs to be further explored. A well-designed large randomized controlled study needs to be conducted in the future. Additionally, patient 2 was not in precisely the same medical condition as patient 1. Patient 2 had coma and was enrolled in the study 1 week after stroke. All clinical scores showed a more severe condition than in patient 1. These factors likely contributed to the limited clinical improvement. More homogeneous cases need to be studied in the future, although the homogeneity of the cases may be difficult to control.

## Conclusion

This study suggests that EEG and fMRI analysis can be used to picture the brain network, identify stimulation targets, and evaluate treatment efficacy. Large clinical trials need to be conducted to test the efficacy of repeated simultaneous tDCS and rTMS in MCS patients.

## Ethics Statement

All clinical data in this case report were provided by the patient’s parents or collected by our team members with the consent of the patient’s parents. The study was approved by the ethics committee of the Xuanwu Hospital, Capital Medical University (ChiCTR1800014293). Written informed consent was obtained from the patient’s parents for participation in the study and publication of this report.

## Author Contributions

YL oversaw data acquisition, reviewed the literature, and drafted the manuscript. TL analyzed and interpreted the EEG data. YW designed the study, supervised the initial drafting, and critically revised the manuscript. QH treated the patient and acquired the clinical data. YS, WC, DG, and HY managed the patient, evaluated the clinical scores, and critically revised the manuscript. XT analyzed and interpreted the EEG data. THu and ZZ analyzed and interpreted the fMRI data. THa treated the patient.

## Conflict of Interest Statement

The authors declare that the research was conducted in the absence of any commercial or financial relationships that could be construed as a potential conflict of interest.

## References

[B1] Angelakis E. Liouta E. Andreadis N. Korfias S. Ktonas P. Stranjalis G. (2014). Transcranial direct current stimulation effects in disorders of consciousness. *Arch. Phys. Med. Rehabil.* 95 283–289. 10.1016/j.apmr.2013.09.002 24035769

[B2] Cavaliere C. Aiello M. Di Perri C. Amico E. Martial C. Thibaut A. (2016). Functional connectivity substrates for tDCS response in minimally conscious state patients. *Front. Cell Neurosci.* 10:257. 10.3389/fncel.2016.00257 27857682 PMC5093112

[B3] Cole M. W. Yarkoni T. Repovs G. Anticevic A. Braver T. S. (2012). Global connectivity of prefrontal cortex predicts cognitive control and intelligence. *J Neurosci.* 32 8988–8999. 10.1523/JNEUROSCI.0536-12.2012 22745498 PMC3392686

[B4] Crone J. S. Soddu A. Holler Y. Vanhaudenhuyse A. Schurz M. Bergmann J. (2014). Altered network properties of the fronto-parietal network and the thalamus in impaired consciousness. *Neuroimage Clin.* 4 240–248. 10.1016/j.nicl.2013.12.005 24455474 PMC3895618

[B5] D’Esposito M. Ballard D. Aguirre G. K. Zarahn E. (1998). Human prefrontal cortex is not specific for working memory: a functional MRI study. *Neuroimage* 8 274–282. 10.1006/nimg.1998.0364 9758741

[B6] Di Perri C. Bahri M. A. Amico E. Thibaut A. Heine L. Antonopoulos G. (2016). Neural correlates of consciousness in patients who have emerged from a minimally conscious state: a cross-sectional multimodal imaging study. *Lancet Neurol* 15 830–842. 10.1016/S1474-4422(16)00111-3 27131917

[B7] Fasoula A. Attal Y. Schwartz D. (2013). Comparative performance evaluation of data-driven causality measures applied to brain networks. *J. Neurosci. Methods* 215 170–189. 10.1016/j.jneumeth.2013.02.021 23537932

[B8] Giacino J. T. Ashwal S. Childs N. Cranford R. Jennett B. Katz D. I. (2002). The minimally conscious state: definition and diagnostic criteria. *Neurology* 58 349–353. 10.1212/wnl.58.3.349 11839831

[B9] Giacino J. T. Kalmar K. Whyte J. (2004). The JFK coma recovery scale-revised: measurement characteristics and diagnostic utility. *Arch. Phys. Med. Rehabil.* 85 2020–2029. 10.1016/j.apmr.2004.02.033 15605342

[B10] Gómez-Herrero G. (2007). *Automatic Artifact Removal (AAR) Toolbox v1.3*. Tampere: Tampere University of Technology.

[B11] Greicius M. D. Krasnow B. Reiss A. L. Menon V. (2003). Functional connectivity in the resting brain: a network analysis of the default mode hypothesis. *Proc. Natl. Acad. Sci. U.S A.* 100 253–258. 10.1073/pnas.0135058100 12506194 PMC140943

[B12] He B. Dai Y. Astolfi L. Babiloni F. Yuan H. Yang L. (2011). eConnectome: A MATLAB toolbox for mapping and imaging of brain functional connectivity. *J. Neurosci. Methods* 195 261–269. 10.1016/j.jneumeth.2010.11.015 21130115 PMC3244474

[B13] Heine L. Soddu A. Gomez F. Vanhaudenhuyse A. Tshibanda L. Thonnard M. (2012). Resting state networks and consciousness: alterations of multiple resting state network connectivity in physiological, pharmacological, and pathological consciousness states. *Front. Psychol.* 3:295. 10.3389/fpsyg.2012.00295 22969735 PMC3427917

[B14] Herwig U. Satrapi P. Schönfeldt-Lecuona C. (2003). Using the international 10-20 EEG system for positioning of transcranial magnetic stimulation. *Brain Topogr.* 16 95–99. 10.1023/b:brat.0000006333.93597.9d 14977202

[B15] Laureys S. Boly M. Maquet P. (2006). Tracking the recovery of consciousness from coma. *J. Clin. Invest.* 116 1823–1825. 10.1172/jci29172 16823480 PMC1483158

[B16] Laureys S. Piret S. Ledoux D. (2005). Quantifying consciousness. *Lancet Neurol.* 4 789–790. 10.1016/s1474-4422(05)70230-1 16297833

[B17] Laureys S. Schiff N. D. (2012). Coma and consciousness: paradigms (re)framed by neuroimaging. *Neuroimage* 61 478–491. 10.1016/j.neuroimage.2011.12.041 22227888

[B18] Leech R. Kamourieh S. Beckmann C. F. Sharp D. J. (2011). Fractionating the default mode network: distinct contributions of the ventral and dorsal posterior cingulate cortex to cognitive control. *J. Neurosci.* 31 3217–3224. 10.1523/JNEUROSCI.5626-10.2011 21368033 PMC6623935

[B19] Lefaucheur J. P. Andre-Obadia N. Antal A. Ayache S. S. Baeken C. Benninger D. H. (2014). Evidence-based guidelines on the therapeutic use of repetitive transcranial magnetic stimulation (rTMS). *Clin. Neurophysiol.* 125 2150–2206. 10.1016/j.clinph.2014.05.021 25034472

[B20] Lefaucheur J. P. Antal A. Ayache S. S. Benninger D. H. Brunelin J. Cogiamanian F. (2017). Evidence-based guidelines on the therapeutic use of transcranial direct current stimulation (tDCS). *Clin. Neurophysiol.* 128 56–92. 10.1016/j.clinph.2016.10.087 27866120

[B21] Lieberman M. D. (2007). Social cognitive neuroscience: a review of core processes. *Annu. Rev. Psychol.* 58 259–289. 10.1146/annurev.psych.58.110405.085654 17002553

[B22] Manganotti P. Formaggio E. Storti S. F. Fiaschi A. Battistin L. Tonin P. (2013). Effect of high-frequency repetitive transcranial magnetic stimulation on brain excitability in severely brain-injured patients in minimally conscious or vegetative state. *Brain Stimul.* 6 913–921. 10.1016/j.brs.2013.06.006 23928101

[B23] Martens G. Lejeune N. O’Brien A. T. Fregni F. Martial C. Wannez S. (2018). Randomized controlled trial of home-based 4-week tDCS in chronic minimally conscious state. *Brain Stimul.* 11 982–990. 10.1016/j.brs.2018.04.021 29759943

[B24] Muller-Dahlhaus F. Ziemann U. (2015). Metaplasticity in human cortex. *Neuroscientist* 21 185–202. 10.1177/1073858414526645 24620008

[B25] Nitsche M. A. Roth A. Kuo M. F. Fischer A. K. Liebetanz D. Lang N. (2007). Timing-dependent modulation of associative plasticity by general network excitability in the human motor cortex. *J. Neurosci.* 27 3807–3812. 10.1523/jneurosci.5348-06.2007 17409245 PMC6672399

[B26] Parlatini V. Radua J. Dell’Acqua F. Leslie A. Simmons A. Murphy D. G. (2017). Functional segregation and integration within fronto-parietal networks. *Neuroimage* 146 367–375. 10.1016/j.neuroimage.2016.08.031 27639357 PMC5312783

[B27] Thibaut A. Bruno M. A. Ledoux D. Demertzi A. Laureys S. (2014). tDCS in patients with disorders of consciousness: sham-controlled randomized double-blind study. *Neurology* 82 1112–1118. 10.1212/wnl.0000000000000260 24574549

[B28] Thibaut A. Wannez S. Donneau A. F. Chatelle C. Gosseries O. Bruno M. A. (2017). Controlled clinical trial of repeated prefrontal tDCS in patients with chronic minimally conscious state. *Brain Inj.* 31 466–474. 10.1080/02699052.2016.1274776 28281845

[B29] Thielscher A. Antunes A. Saturnino G. (2015). “Field modeling for transcranial magnetic stimulation: a useful tool to understand the physiological effects of TMS? Engineering in Medicine and Biology Society (EMBC),” in *37th Annual International Conference of the IEEE*, (Milan). 10.1109/EMBC.2015.7318340 26736240

[B30] Tian L. Jiang T. Liu Y. Yu C. Wang K. Zhou Y. (2007). The relationship within and between the extrinsic and intrinsic systems indicated by resting state correlational patterns of sensory cortices. *Neuroimage* 36 684–690. 10.1016/j.neuroimage.2007.03.044 17499519

[B31] Vanhaudenhuyse A. Demertzi A. Schabus M. Noirhomme Q. Bredart S. Boly M. (2011). Two distinct neuronal networks mediate the awareness of environment and of self. *J. Cogn. Neurosci.* 23 570–578. 10.1162/jocn.2010.21488 20515407

[B32] Vanhaudenhuyse A. Noirhomme Q. Tshibanda L. J. Bruno M. A. Boveroux P. Schnakers C. (2010). Default network connectivity reflects the level of consciousness in non-communicative brain-damaged patients. *Brain* 133 161–171. 10.1093/brain/awp313 20034928 PMC2801329

[B33] Wang X. Zhen Z. Song Y. Huang L. Kong X. Liu J. (2016). The hierarchical structure of the face network revealed by its functional connectivity pattern. *J Neurosci.* 36 890–900. 10.1523/JNEUROSCI.2789-15.2016 26791218 PMC6601995

[B34] Wilke C. Worrell G. He B. (2011). Graph analysis of epileptogenic networks in human partial epilepsy. *Epilepsia* 52 84–93. 10.1111/j.1528-1167.2010.02785.x 21126244 PMC3200119

[B35] Yeo B. T. Krienen F. M. Sepulcre J. Sabuncu M. R. Lashkari D. Hollinshead M. (2011). The organization of the human cerebral cortex estimated by intrinsic functional connectivity. *J Neurophysiol.* 106 1125–1165. 10.1152/jn.00338.2011 21653723 PMC3174820

[B36] Zhang D. Zhao H. Bai W. Tian X. (2016). Functional connectivity among multi-channel EEGs when working memory load reaches the capacity. *Brain Res.* 1631 101–112. 10.1016/j.brainres.2015.11.036 26638838

