# Supplementary material for: Electroencephalography and Functional Magnetic Resonance Imaging-Guided Simultaneous Transcranial Direct Current Stimulation and Repetitive Transcranial Magnetic Stimulation in a Patient With Minimally Conscious State
Source: Front Neurosci. 2019 Jul 31;13:746. doi: 10.3389/fnins.2019.00746 (PMC6685103; doi:10.3389/fnins.2019.00746)
Supplement: Supplementary file 1 [file Data_Sheet_1.pdf]

Supplementary Material

Con	Sti		Sham	
	MCS_pre	MCS_post	MCS_pre	MCS_post
5	3	2	3	3
5	3	2	3	3
5	3	3	3	3
5	3	3	3	3
5	3	3	3	3
5	3	3	3	3
5	3	3	3	3
5	3	3	3	3
5	3	3	3	3
5	3	3	3	3
5	5	3	3	3
5	5	3	3	3
5	5	3	3	3
5	5	5	3	3
5	5	5	3	3
	5	5	3	3
	5	5	3	
	5	5	3	
	5	5	3	
	5	5	3	
	5	5	3	
	5	5	3	
	5		5	
	5		5	
	5		5	
			5	
			5	
			5	
			5	
			5	

Supplementary Table 1. MVAR model order of the patients and healthy controls. The numbers of segments in the different groups differ. The orders are not consistent across patients and healthy controls.

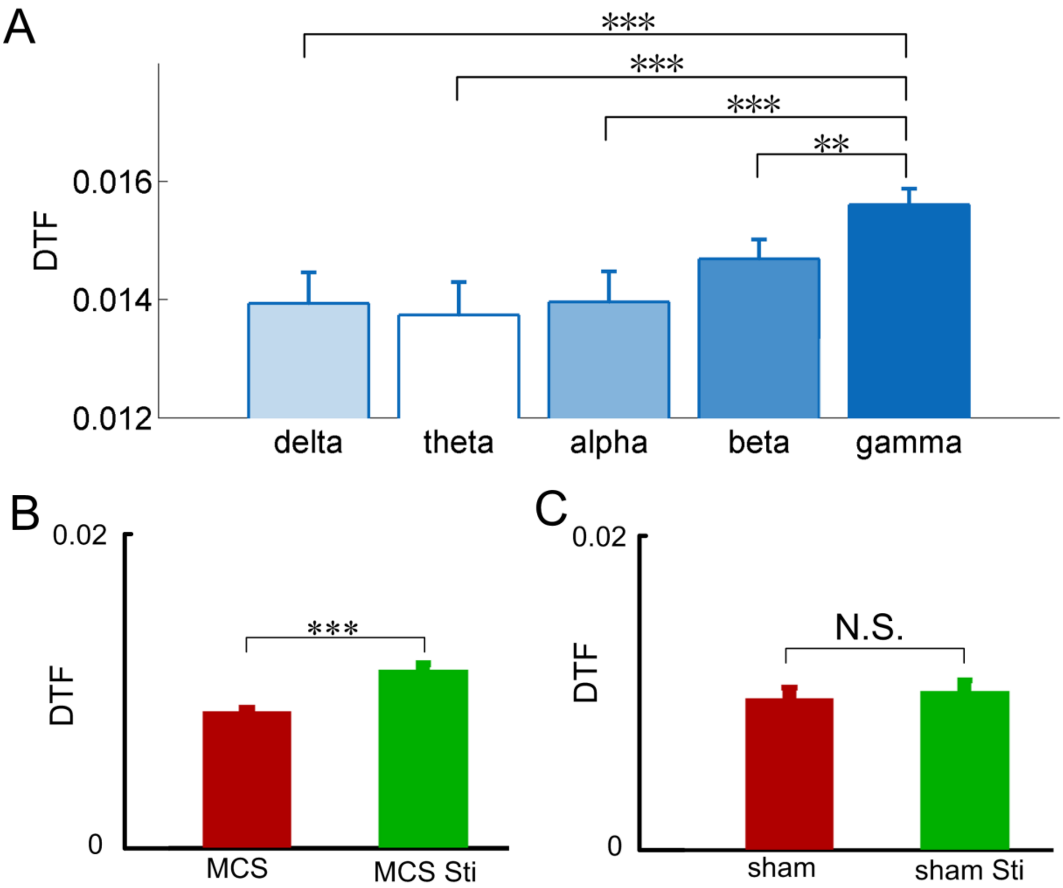

Supplementary Figure 1. Statistical comparison of the DTF values across different frequency ranges, pre- and post-stimulation. (A) Gamma range DTF shows a significant difference (ANOVA, $F = 14.254$, $p < 0.001$; *post hoc*, **$p < 0.01$, ***$p < 0.001$). Data in the figure are expressed as the mean ± SEM. (B) The DTF values in gamma shows the significant statistical difference between pre- and post-stimulation in MCS (***$p < 0.001$). (C) The DTF values in gamma do not show difference between pre- and post-treatment in patient 2 ($p > 0.05$). DTF, directional transfer function; MCS, minimally conscious state; MCS Sti, minimally conscious state after treatment; N.S., not significant; Pt 2, patient 2; Pt 2 post, patient 2 after treatment.
